# Structural and Functional Characterization of Two Alternative Splicing Variants of Mouse Endothelial Cell-Specific Chemotaxis Regulator (ECSCR)

**DOI:** 10.3390/ijms13044920

**Published:** 2012-04-19

**Authors:** Wen Wu, Chunwei Shi, Fanxin Ma, James Balducci, Hanju Huang, Hong-Long Ji, Yongchang Chang, Yao Huang

**Affiliations:** 1 Department of Obstetrics and Gynecology, St. Joseph’s Hospital and Medical Center, Phoenix, AZ 85004, USA; E-Mails: wuwen19821013@hotmail.com (W.W.); scwoycj@yahoo.com.cn (C.S.); fanxin.ma@gmail.com (F.M.); james.balducci@chw.edu (J.B.); 2 Barrow Neurological Institute, St. Joseph’s Hospital and Medical Center, Phoenix, AZ 85013, USA; E-Mail: yongchang.chang@chw.edu; 3 Department of Pathogen Biology, Tongji Medical College, Huazhong University of Science and Technology, Wuhan, Hubei 430030, China; E-Mail: juguangying@yahoo.com.cn; 4 Department of Biochemistry, University of Texas Health Science Center at Tyler, Tyler, TX 75708, USA; E-Mail: HongLong.Ji@uthct.edu

**Keywords:** ECSCR/ECSM2, alternative splicing, isoform, gene structure, exon-intron boundary, cDNA cloning and expression, endothelial cell migration

## Abstract

Endothelial cells (ECs) that line the lumen of blood vessels are important players in blood vessel formation, and EC migration is a key component of the angiogenic process. Thus, identification of genes that are specifically or preferentially expressed in vascular ECs and in-depth understanding of their biological functions may lead to discovery of new therapeutic targets. We have previously reported molecular characterization of human endothelial cell-specific molecule 2 (ECSM2)/endothelial cell-specific chemotaxis regulator (ECSCR). In the present study, we cloned two mouse full-length cDNAs by RT-PCR, which encode two putative ECSCR isoform precursors with considerable homology to the human ECSCR. Nucleotide sequence and exon-intron junction analyses suggested that they are alternative splicing variants (ECSCR isoform-1 and -2), differing from each other in the first and second exons. Quantitative RT-PCR results revealed that isoform-2 is the predominant form, which was most abundant in heart, lung, and muscles, and moderately abundant in uterus and testis. In contrast, the expression of isoform-1 seemed to be more enriched in testis. To further explore their potential cellular functions, we expressed GFP- and FLAG-tagged ECSCR isoforms, respectively, in an ECSCR deficient cell line (HEK293). Interestingly, the actual sizes of either ECSCR-GFP or -FLAG fusion proteins detected by immunoblotting are much larger than their predicted sizes, suggesting that both isoforms are glycoproteins. Fluorescence microscopy revealed that both ECSCR isoforms are localized at the cell surface, which is consistent with the structural prediction. Finally, we performed cell migration assays using mouse endothelial MS1 cells overexpressing GFP alone, isoform-1-GFP, and isoform-2-GFP, respectively. Our results showed that both isoforms significantly inhibited vascular epidermal growth factor (VEGF)-induced cell migration. Taken together, we have provided several lines of experimental evidence that two mouse ECSCR splicing variants/isoform precursors exist. They are differentially expressed in a variety of tissue types and likely involved in modulation of vascular EC migration. We have also defined the gene structure of mouse ECSCR using bioinformatics tools, which provides new information towards a better understanding of alternative splicing of ECSCR.

## 1. Introduction

Angiogenesis or neovascularization is a physiological process by which new blood vessels develop from pre-existing vasculature [1]. It is not only fundamental for normal organ growth and development, wound healing, and female reproductive functions, but also critically involved in many pathological conditions, such as ischemic vascular diseases, atherosclerosis, tumor growth and metastasis, rheumatoid arthritis, diabetic retinopathies, and age-related macular degeneration [2–5]. Endothelial cells (ECs) constitute the inner layer of blood vessels and are the important players in angiogenesis, and EC migration is a key step during angiogenic process [6]. Thus, there has been a great deal of interest in identifying new genes specifically or preferentially expressed in vascular endothelial cells and studying their biological functions, which will help to develop more specific and effective pro- and anti-angiogenesis therapies.

Over the past several decades, massive efforts towards this direction have resulted in the identification of a number of important EC-specific or EC-highly expressed molecules, such as platelet endothelial cell adhesion molecule (PECAM-1 or CD31) [7], vascular endothelial cell adhesion molecule-1 (VCAM-1) [8], endothelial cell-selective adhesion molecule (ESAM) [9], vascular endothelial (VE)-cadherin (CD144 or cadherin 5) [10], vascular endothelial growth factor (VEGF) [11], *etc*. Newly developed bioinformatics strategies, such as database mining and virtual screening of public gene libraries, combined with transcriptional profiling (e.g., microarray and reverse transcription PCR) have been employed to “clone” more novel EC-specific genes [12,13]. One of these important genes is human endothelial cell-specific molecule 2 (*ECSM2*) [14,15]. This gene has recently been described as apoptosis regulator through modulating cIAP expression (*ARIA*) [16,17]. Currently, its approved nomenclature by the National Center for Biotechnology Information (NCBI) is endothelial cell-specific chemotaxis receptor (*ECSCR*) [18,19]. Despite its discovery about a decade ago, the biological and cellular functions of ECSCR/ECSM2/ARIA have only recently begun to be understood. Our laboratory is one of the two research groups who independently reported the molecular characteristics and cellular functions of human ECSCR gene [14,15]. Most recently, we have demonstrated that ECSCR can localize to cell-cell junction and modulate basic fibroblast growth factor (bFGF)-directed cell migration [20].

Alternative splicing of pre-mRNA is a common posttranscriptional process utilized by eukaryotic organisms to generate multiple transcript variants from a single gene [21]. Alternatively spliced exons have splice sites that can be specifically recognized depending on tissue type, developmental stage, external stimuli, cellular stress, or pathological conditions [22]. Thus, alternative splicing has been considered as a major mechanism contributing to transcriptome and proteome complexity [23,24]. In this study, we cloned two ECSCR full-length cDNA variants from mouse blood vessels, which likely encode two different hypothetical protein products of a single ECSCR gene. We named them ECSCR isoform-1 and isoform-2, respectively. Furthermore, we characterized the molecular structure and function of the two isoforms using combined bioinformatics and experimental approaches.

## 2. Experimental Section

### 2.1. Reagents and Antibodies

Recombinant human VEGF and 4′,6-diamidino-2-phenylindole dihydrochloride (DAPI) were purchased from Sigma (St. Louis, MO, USA). One kilobase DNA ladder and 1 kb Plus DNA ladder were obtained from Invitrogen (Carlsbad, CA, USA). All routine chemicals and reagents were from Sigma or Fisher Scientific (Pittsburgh, PA, USA) unless otherwise noted. Anti-GFP polyclonal antibody and anti-FLAG monoclonal antibody M2 were obtained from Sigma. Tetramethyl Rhodamine Isothiocyanate (TRITC)-conjugated goat anti-mouse IgG secondary antibody was from Jackson ImmunoResearch Laboratories (West Grove, PA, USA). Horseradish peroxidase (HRP)-conjugated goat anti-rabbit and goat anti-mouse secondary antibodies were purchased from Pierce (Rockford, IL, USA).

### 2.2. RNA Extraction, RT-PCR, and Quantitative Real-Time PCR

Total RNAs were extracted from mouse large blood vessels (aorta) and other tissue types using TRIzol Reagent (Invitrogen). cDNA synthesis was performed with SuperScript III First-strand Synthesis Supermix (Invitrogen) and PCR was carried out using Pfu Ultra DNA polymerase (Agilent Technologies, Santa Clara, CA, USA). Quantitative PCR was performed using Platinum SYBR Green qPCR Supermix UDG Kit (Invitrogen) on the iQ5 Real-Time PCR Detection System (Bio-Rad, Hercules, CA, USA). The mRNA level of ECSCR was normalized to that of the mouse housekeeping genes, β-actin and GAPDH, respectively.

### 2.3. Plasmid Constructs and Sequencing

Full-length cDNAs encoding mouse ECSCR isoform-1 and isoform-2 precursors, respectively, were obtained by RT-PCR and cloned into the expression vectors, pEGFP-N1 (Clontech Laboratories, Palo Alto, CA, USA) and p3×FLAG-CMV-14 (Sigma), respectively, as previously described [14,25,26]. These recombinant plasmid constructs were confirmed by sequencing, and named pECSCR isoform-1-GFP, pECSCR isoform-2-GFP, pECSCR isoform-1-FLAG, and pECSCR isoform-2-FLAG, respectively.

### 2.4. Cell Culture and Transfection

Human kidney epithelial (HEK) 293 cells and mouse endothelial MS1 cells (both from ATCC, Manassas, VA, USA) were grown in Dulbecco’s modified Eagle’s medium (DMEM) (Mediatech, Manassas, VA, USA) containing 10% fetal bovine serum (FBS) (Sigma), 100 units/mL penicillin, and 100 μg/mL streptomycin (both from Mediatech). Plasmid DNAs were transiently transfected into these two cell lines using Lipofectamine 2000 (Invitrogen), as described previously [14,25,26]. Transfection efficiency was confirmed by immunoblotting and/or immunofluorescent microscopy. The transfectants were subjected to various assays 48 h post transfection.

### 2.5. Protein Extraction and Western Blot Analysis (Immunoblotting)

Protein extraction from cells, quantitation, and Western blot analysis (WB) were performed as described elsewhere [27–29]. Briefly, cells were washed once with ice-cold phosphate-buffered saline (PBS) and then harvested by scraping in PBS followed by centrifugation. The cell pellets were lysed and solubilized in lysis buffer (50 mM Tris-HCl (pH 8.0), 2 mM EDTA, 150 mM NaCl, 100 mM NaF, 10% glycerol, 1% SDS, 1 mM phenylmethylsulfony fluoride, 5 μg/mL aprotinin, and 5 μg/mL leupeptin). The cell extracts were quantitated with bicinchoninic acid (BCA) reagents (Pierce) and used for Western blot analysis with antibodies as specified in each experiment. Immunoblotting signals were detected with SuperSignal chemiluminescent substrate (Pierce) and digital images were captured using a Kodak 4000 MM molecular imager.

### 2.6. Immunofluorescent Staining and Microscopy

Cells were grown on glass coverslips precoated with gelatin (2%) and fixed in 4% paraformaldehyde for 15 min. When applicable, the cells were permeabilized with 0.25% Triton X-100 in PBS containing 1% bovine serum albumin (BSA) for 15 min and stained with mouse anti-FLAG monoclonal antibody M2. The mouse antibody was detected with TRITC-conjugated goat anti-mouse IgG secondary antibody. The coverslips were mounted onto microscope slides in Vectashield mounting medium for fluorescence containing DAPI (Vector Laboratories, Burlingame, CA). The cells expressing GFP alone or GFP fusion proteins were directly used for fluorescent visualization without antibody staining. Fluorescent images were captured with a Zeiss Axio Imager upright fluorescent microscope (Carl Zeiss, Thornwood, NY, USA), as described previously [20,25].

### 2.7. Cell Migration Assay

MS1 cells were transiently transfected with pECSCR isoform-1-GFP, pECSCR isoform-2-GFP, or pEGFP-N1 (vector control) for 48 h. The transfectants were subjected to Transwell migration assay as described previously [20,28]. Briefly, BD Falcon 8 μm pore inserts (BD Biosciences, San Diego, CA, USA) were placed in a 12-well plate containing 1 mL of DMEM medium containing low serum (1% FBS) and VEGF (10 ng/mL) (lower chamber). Single cell suspensions (2.5 × 10^5^ cells in 0.5 mL of DMEM containing 1% FBS per well) were added to the upper chamber of each Transwell insert and incubated at 37 °C for 5 h. At the end of incubation, the cells were fixed by submerging the Transwell inserts in 4% paraformaldehyde and counterstained with DAPI (0.5 μg/mL). The non-migrated cells on the top side of the membrane were removed with wet cotton swabs. Air dried membranes were cut out from the Transwell inserts, mounted onto microscope slides, and examined using a Zeiss Axio Imager upright fluorescent microscope (Carl Zeiss). The number of cells migrated across the membranes per imaging field was counted (*n* = 10 per condition).

### 2.8. Statistical Analysis

All statistical data were from multiple measurements as specified in each experiment and presented as mean ± SD. The significance of differences was estimated using unpaired Student’s *t*-test and *p* < 0.05 was considered significant.

### 2.9. Protein-Protein Basic Local Alignment Search Tool (BLASTP)

The full-length protein sequence of human ECSCR (205 amino acids) was extracted from the GenBank via the NCBI website and used as a query sequence to blast the NCBI non-redundant protein sequences (nr), including all non-redundant GenBank CDS translations + PDB + SwissProt + PIR + PRF excluding environmental samples from whole genome shotgun (WGS) sequencing projects, using BLASTP 2.2.26+ program [30,31].

### 2.10. Multiple Sequence Alignment

Alignments of multiple nucleotide or amino acid sequences were made using the ClustalW2 program via the EMBL-EBI website [32,33].

### 2.11. Sequence Translation

Translation of a DNA sequence to a protein sequence was performed using the ExPASy Translate tool [34].

### 2.12. Prediction of Signal Peptide and Transmembrane Domain

Prediction of signal peptide was made using several web-based programs including SIG-Pred [35], PrediSi [36], and SignalP 4.0 [37]. Transmembrane domain was predicted using the Transmembrane Prediction Server (DAS) [38,39].

### 2.13. Prediction of Glycosylation Sites

Putative *N*-glycosylation and *O*-glycosylation sites were predicted using NetNGlyc 1.0 Server [40] and NetOGlyc 3.1 server [41,42], respectively.

## 3. Results and Discussion

**Figure 1:**
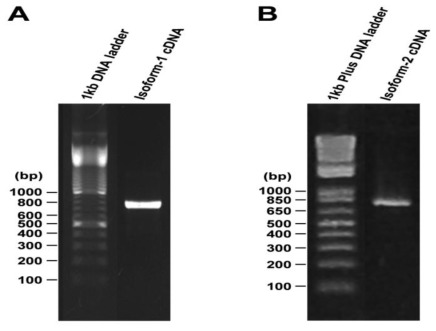
RT-PCR to obtain full-length cDNAs encoding mouse ECSCR isoform-1 and isoform-2 precursors. Total RNAs were extracted from mouse large blood vessels (aorta) and RT-PCR was performed to obtain full-length cDNAs that encode mouse ECSCR isoform-1 (**A**) and isoform-2 (**B**), respectively. DNA ladders are shown.

In our previous study, we identified a number of hypothetical proteins across species exhibiting substantial sequence homology to the human ECSCR/ECSM2 (GenBank acc No. NP_001071161), one of which is mouse ECSCR (GenBank acc No. NP_001028313) [14]. To explore the possibility of existence of multiple isoforms of ECSCR, here we performed a protein-protein blast search (BLASTP) on non-redundant protein sequences using the 205-amino acid human ECSCR (Swiss-Prot acc No. Q19T08) as a query sequence. Interestingly, we obtained two hits from the mouse species. One is annotated as ECSCR/ECSM2 (GenBank acc No. NP_001028313.1), as expected, and the other one is RIKEN cDNA 1110006O17 (GenBank acc No. EDK97141.1). We noticed that the two hypothetical proteins only differ in the first 30–35 amino acids at their *N*-termini. Given that the mouse ECSCR sequence with GenBank acc No. NP_001028313.1 [43] has been reported in several recent literatures [14,16,17,19,44,45], here we designated it as ECSCR isoform-1 precursor and the RIKEN cDNA 1110006O17 (GenBank acc No. EDK97141.1) as isoform-2 precursor, respectively. Based on the available information of their corresponding nucleotide sequences in the NCBI databases, we designed specific primers for both isoforms and obtained full-length of cDNAs by RT-PCR using total RNAs extracted from mouse large blood vessels (aorta) (Figure 1). The open reading frames of the two cDNAs and their alignment results are shown in Figures S1–S3. The coding regions of the two transcripts are nearly identical except for the initial ~100 bp segments (Figure S3), suggesting that they are alternative splicing variants.

**Figure 2:**
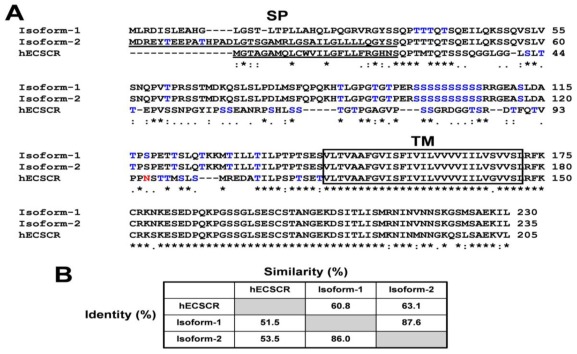
Identification of mouse ECSCR isoform-1 and isoform-2 precursors. (**A**) Amino acid sequence alignment of mouse ECSCR isoform-1, isoform-2, and human ECSCR precursors. Putative signal peptides (SP) are underlined and a single transmembrane domain (TM) is boxed. Predicted *N*-glycosylation and *O*-glycosylation sites are shown in red and blue, respectively. (**B**) Percentages of identity and similarity among the amino acid sequences of mouse ECSCR isoform-1, isoform-2, and human ECSCR precursors.

Alignment of amino acid sequences of human ECSCR and the two mouse isoform precursors is displayed in Figure 2A. We also performed structure prediction using the ExPASy proteomic tools [46]. The results indicated that, similar to the human ECSCR, both mouse isoforms are most likely membrane proteins, each containing a long *N*-terminal extracellular domain (ECD), a single transmembrane domain (TM), and a short intracellular domain (ICD) at the *C*-terminus (Figure 2A). Using the SIG-Pred (Signal Peptide Prediction) program [35], the human ECSCR and the mouse ECSCR isoform-2 precursors were predicted to contain a signal peptide (SP) of 24 and 39 amino acid residues at their respective *N*-terminus with a high score (Figure 2A). In contrast, no signal peptide was predicted for the mouse isoform-1 precursor due to an extremely low score. Similar results were obtained when other signal peptide prediction programs, such as PrediSi [36] and SignalP 4.0 Server [37], were employed. Furthermore, we used NetNGlyc 1.0 Server [40] and NetOGlyc 3.1 server [41] to search for potential *N*-glycosylation sites (Asn-Xaa-Ser/Thr sequons) and mucin-type *O*-glycosylation Ser/Thr sites, respectively. The human ECSCR has a putative *N*-glycosylation site within its *N*-terminal ECD whereas no *N*-glycosylation site was found in the two mouse ECSCR sequences (Figure 2A). In contrast, a number of potential *O*-glycosylation sites were identified in all three ECSCR proteins as indicated in Figure 2A. We also noted that the predicted *O*-glycosylation sites of mouse ECSCR isoform-1 slightly differ from those of isoform-2 (Figure 2A). The sequence homology (identify and similarity) among these proteins is shown in Figure 2B.

**Figure 3:**
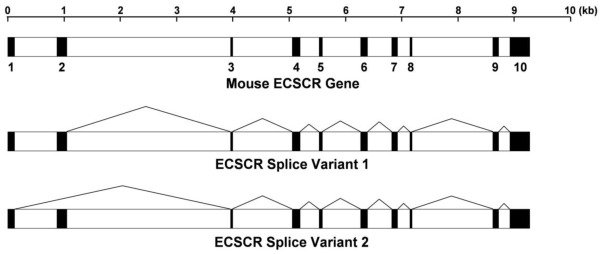
Gene structure of mouse ECSCR and alternative splicing events. The reconstructed mouse ECSCR gene is based on new data presented in this study. A total of ten exons are indicated by closed boxes.

To further define the gene structure encoding the two mouse ECSCR isoform precursors, we examined the mouse genome sequences and successfully mapped the two cDNAs/transcripts within the region from 19601084 to 19610340 of *Mus musculus* 181000113601716 genomic scaffold, whole genome shotgun sequence [47]. We identified a total of 10 exons, of which Exon 1 is spliced out in isoform-1 and Exon 2 is spliced out in isoform-2 (Figure 3). The updated mouse ECSCR gene sequences are presented in Figure S4, in which all ten exons and exon-intron boundaries are marked. We noted that in the current public annotation for mRNA product “RIKEN cDNA 1110006O17” (referred to as isoform-2 here) [47], only nine exons are defined, which do not include the definition of Exon 2. Our results clearly indicated that the mouse ECSCR isoform-1 and isoform-2 precursors are indeed splicing variants from a single *ECSCR* gene.

**Figure 4:**
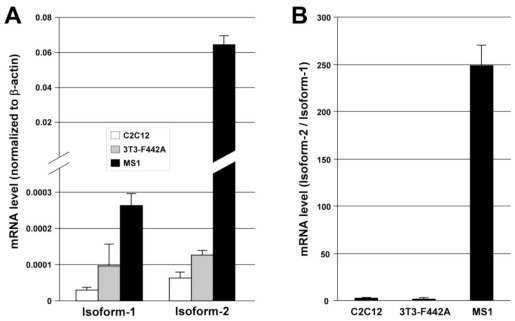
Expression of ECSCR isoform-1 and isoform-2 in cultured mouse cell lines. (**A**) The mRNA levels of isoform-1 and isoform-2 measured by qRT-PCR. C2C12, mouse myoblasts; 3T3-F442A, mouse preadipocytes; MS1, mouse endothelial cells. (**B**) The ratio of mRNA level of isoform-2 to that of isoform-1 indicates that isoform-2 is the predominant form expressed in endothelial MS1 cells. Data are mean ± SD (*n* = 3).

**Figure 5:**
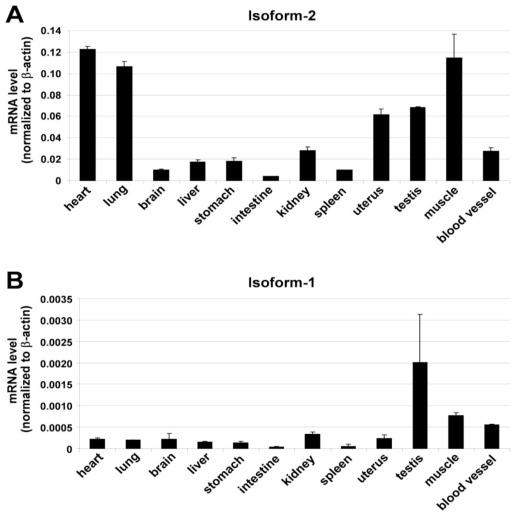
Expression of ECSCR isoform-1 and isoform-2 in a variety of mouse tissues. The mRNA levels of isoform-2 (**A**) and isoform-1 (**B**) measured by qRT-PCR. Blood vessels examined here were large vessels (aorta). Data are mean ± SD (*n* = 3).

We next sought to compare the expression patterns of the two splicing variants in a variety of mouse cell lines and tissues by quantitative real-time RT-PCR (qRT-PCR). As shown in Figure 4A,B, when normalized to the expression level of a housekeeping gene (β-actin), both ECSCR isoform-1 and isoform-2 were highly expressed in the mouse EC line (MS1) but not in the non-EC lines (C2C12 and 3T3-F442A) tested here. Noticeably, however, the abundance of transcripts of isoform-2 was approximately 200 fold of that of isoform-1 (Figure 4B), suggesting that isoform-2 is the predominant form expressed in mouse ECs. Furthermore, our qRT-PCR results indicated that both isoforms were differentially expressed among a variety of tissues examined here (Figure 5). The transcripts of isoform-2 were most abundant in heart, lung, and muscles, and moderately abundant in uterus and testis. Surprisingly, its expression level in the above-mentioned tissues was higher than that in large blood vessels (aorta) (Figure 5A). As for isoform-1, its transcripts were found to be relatively high in testis, muscles, and large blood vessels (Figure 5B). Regardless of the tissue types, in general, the expression level of isoform-2 mRNA was much higher than that of isoform-1. For example, the abundance of isoform-2 transcripts was ~150 fold of that of isoform-1 in muscles and ~50 fold in large blood vessels (aorta). Similar results were obtained when the expression levels of isoform-1 and isoform-2 mRNA were normalized to another commonly used housekeeping gene, glyceraldehyde 3-phosphate dehydrogenase (GAPDH) (data not shown).

ECs line the circulatory system, including the heart, arteries, veins and capillaries. It is now recognized that ECs have important physiological functions not only for the circulatory system but also for different organs that the blood vessel serve. In general, ECs can be classified into three categories, including continuous ECs, fenestrated ECs, and discontinuous ECs. The continuous ECs occur in all major blood vessels and capillaries of the lung, heart, muscles, and brain. These ECs are connected by a series of cell-cell junctions. The fenestrated ECs are found in the gastrointestinal villi, glomeruli of kidneys and in capillaries that supply the endocrine glands. Their cell junctions are similar to those of continuous endothelium, but the presence of fenestrate occurs. In contrast, discontinuous ECs are not connected to each other and are often interspersed by another type of cells. The capillaries with discontinuous ECs are known as sinusoidal capillaries, which occur in bone marrow, the spleen and the liver. Thus, the current discovery of higher expression level of ECSCR isoform-2 in the heart, lung, muscles, uterus, and testis than in large blood vessels (Figure 5A) could suggest that isoform-2 is preferentially expressed in capillaries consisting of continuous ECs. Interestingly, this is consistent with our recent findings that ECSCR is a novel EC junctional protein [20]. Based on our experimental results shown in Figures 4 and 5, we concluded that isoform-2 is probably the most common form of ECSCR splicing variants identified so far in the mouse species and it is highly expressed in continuous endothelium (more dominant in capillaries than in large vessels). However, the relatively high abundance of isoform-1 detected in the testis compared to other tissue types may have its biological and pathological significance, which deserves further investigation in the future.

**Figure 6:**
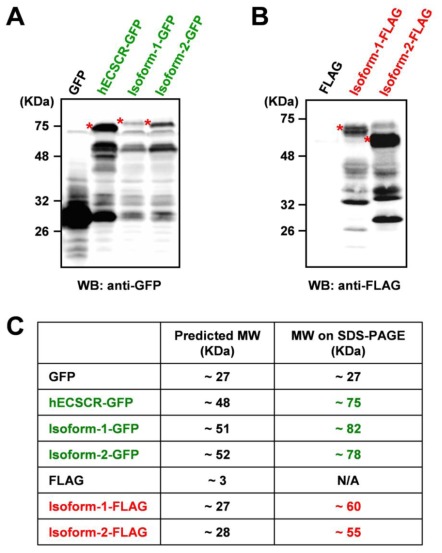
Heterologous expression of mouse ECSCR isoform-1 and isoform-2 proteins suggests that they are likely glycoproteins. (**A** and **B**) GFP- or FLAG-tagged human (h) ECSCR, mouse isoform-1, and isoform-2 were expressed I HEK293 cells, respectively. The expression of fusion proteins was confirmed by Western blot (WB) analysis with anti-GFP (A) or anti-FLAG (B) antibody. The glycosylated (mature) forms of fusion proteins are indicated by asterisks. (**C**) Comparison of the actual molecular weights (MW) of the fusion proteins in kilodaltons (kDa) on SDS-PAGE with their predicted molecular weights (MW) based amino acid sequences.

**Figure 7:**
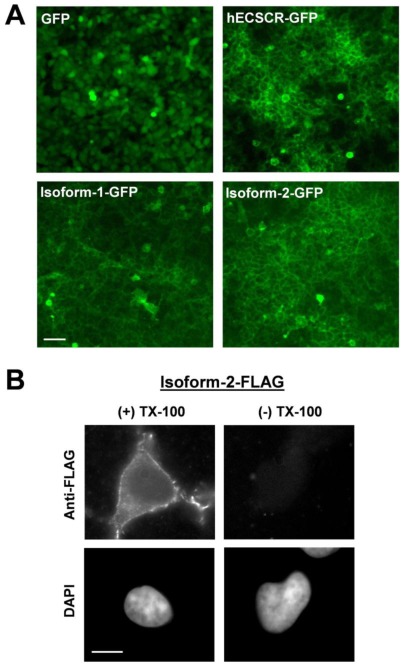
Cell surface localization of mouse ECSCR isoforms. (**A**) HEK293 cells expressing GFP alone, human (h) ECSCR-GFP, mouse ECSCR isoform-1-GFP, and isoform-2-GFP, respectively, were visualized by fluorescent microscopy. Scale bar, 50 μm. (**B**) Mouse ECSCR isoform-2 tagged with FLAG at its *C*-terminus was expressed in HEK293 cells and the transfectants were costained with anti-FLAG antibody and DAPI. The cell surface localization of ECSCR-FLAG was detected only in the cells that were permeabilized with 0.1% Triton X-100. Scale bar, 20 μm.

To further characterize the two mouse ECSCR isoforms at the cellular level, we heterologously expressed both isoforms tagged with either green fluorescent protein (GFP) or FLAG at their *C*-termini in human embryonic kidney (HEK) 293 cell line that does not endogenously express ECSCR [14]. Similarly to what we have previously reported for human ECSCR [14,20], immunoblotting with anti-GFP (Figure 6A) and anti-FLAG (Figure 6B) antibodies, respectively, indicated that the molecular mass of both isoform-1 and isoform-2 was markedly larger than their corresponding predicted sizes (Figure 6C). This suggested that glycosylation may occur during the protein process for both isoforms *in vivo*, as we demonstrated previously for human ECSCR [14,20]. Notably, the actual molecular mass of the mouse ECSCR isoform-1-GFP or isoform-1-FLAG was slightly larger than that of isoform-2-GFP or isoform-2-FLAG on SDS-PAGE (Figure 6A,B). This could be due to the different predicted *O*-glycosylation sites of the two isoforms and/or the absence of a predicted signal peptide in isoform-1 precursor (Figure 2A). Furthermore, like human ECSCR-GFP, both isoform-1-GFP and isoform-2-GFP proteins clearly displayed plasma membrane localization, revealed by fluorescence microscopy (Figure 7A). In particular, immunofluorescent staining using an anti-FLAG antibody demonstrated that membrane-bound mouse ECSCR isoform-2-FLAG was detected only in detergent-permeabilized cells but not in nondetergent-treated cells (Figure 7B), indicating that the *C*-terminus of ECSCR is located in the cytosol. All these results are consistent with our molecular prediction (Figure 2A).

**Figure 8:**
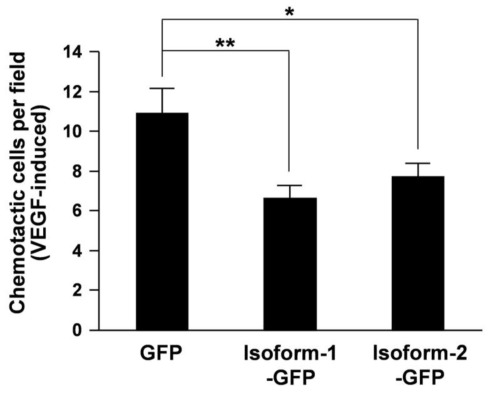
Overexpression of mouse ECSCR isoforms in MS1 cells inhibits endothelial cell motility. MS1 cells overexpressing GFP alone, mouse ECSCR isoform-1-GFP, and isoform-2-GFP, respectively, were subjected to Transwell migration assay, in which VEGF (10 ng/mL) was used as a chemoattractant as detailed in Methods. The cells were allowed to migrate for 5 h. The number of migrated cells per imaging field for each condition was counted. Data are mean ± SD (*n* = 10), * *p* < 0.05, ** *p* < 0.01.

Finally, we explored the potential cellular functions of the two mouse ECSCR isoforms. Here, we used a similar strategy as described previously for human ECSCR [14,20], and assessed VEGF-driven cell migration (chemotaxis) in MS1 cells overexpressing GFP alone, mouse ECSCR isoform-1-GFP, and isoform-2-GFP, respectively. As previously shown, overexpression of GFP in MS1 cells did not affect cell migration [20], which served as a control in the migration assays. In a representative experiment shown in Figure 8, overexpression of either mouse ECSCR isoform-1-GFP or isoform-2-GFP in MS1 cells significantly attenuated VEGF-induced cell motility compared to the GFP control cells. This is in agreement with our previous conclusion that overexpression of human ECSCR inhibits cell migration mediated by growth factors such as epidermal growth factor (EGF) [14] and bFGF [20].

We and other groups have demonstrated that both human ECSCR and mouse ECSCR are critically involved in the modulation of EC migration, a key step during angiogenic process [14,15,17,18,20] (and this study). However, the existing data regarding ECSCR functioning in EC motility and endothelial tube formation on Matrigel (*in vitro* angiogenesis) have shown some discrepancy. Armstrong *et al.* reported that siRNA knockdown of ECSCR in human umbilical vein endothelial cells (HUVECs) inhibited EC migration when fetal calf serum (FCS) and endothelial cell growth supplements (TCS) together were used as the chemoattractant and impaired tube formation [15]. Verma *et al*. showed that knockdown of ECSCR in HUVECs resulted in reduced migration in response to fetal bovine serum (FBS) or VEGF [18]. In contrast, our gain or loss of function assays showed that overexpression or knockdown of human ECSCR in ECs attenuated or enhanced bFGF-directed migration, respectively [20]. Our early work also revealed that coexpression of human ECSCR and EGF receptor (EGFR) in HEK293 cells inhibited EGF-induced cell motility [14]. In the current study, we extended our findings to mouse homologues of ECSCR including two isoforms. Consistent with our previous findings, overexpression of either mouse ECSCR isoform-1 or isoform-2 in MS1 cells resulted in reduced cell migration driven by VEGF. Interestingly, Ikeda *et al*. initially observed that knockdown of ARIA/ECSCR in HUVECs by siRNA reduced endothelial apoptosis without affecting cell migration [16]. However, *in vivo* angiogenesis, which was studied using Matrigel-plug assay, mouse ischemic retinopathy model, and tumor xenograft model, was significantly enhanced by ARIA/ECSCR knockdown [16]. More recently, the work from the same group demonstrated that siRNA-mediated silencing of ARIA/ECSCR in cord blood late outgrowth ECs (OECs) attenuated apoptosis and accelerated migration towards stromal cell-derived factor-1α (SDF-1α), and re-expression of ARIA (ECSCR) reversed the effects of siRNA silencing on apoptosis and migration [17]. They pointed out that the different effects of ARIA/ECSCR knockdown on cell motility from their two studies was probably due to the insufficient silencing in HUVECs (~60% reduction in mRNA) *versus* nearly complete knockout in OECs (~98% reduction in mRNA) [17]. Nevertheless, their later study confirmed that ECs isolated from ARIA/ECSCR knockout mouse aorta exhibited reduced apoptosis and accelerated motility and tube formation [17]. Although it is not clear whether ECSCR isoform-1, isoform-2 or both were knocked out in their mice (generated by targeted deletion of the first three exons of the gene [17]), this piece of elegant work at least suggests that mouse ECSCR has an anti-angiogenic rather than a pro-angiogenic effect. Apparently, our previous and current *in vitro* results strongly support such an opinion.

## 4. Conclusions

In this study, we have provided several lines of experimental evidence that two mouse ECSCR splicing variants (isoform-1 and isoform-2) exist, which are differentially expressed in a variety of tissue types and likely involved in the modulation of vascular EC migration. We have also defined the gene structure of mouse ECSCR using bioinformatics tools, which provides new information towards a better understanding of alternative splicing of ECSCR. The exact functions and, in particular, differential functions of the two isoforms deserve further investigation. This may require successful generation of specific antibodies targeting different ECSCR isoforms, which could be a new challenge in the field.

## Supplementary Materials
