# Supplementary material for: Structural and Functional Characterization of Two Alternative Splicing Variants of Mouse Endothelial Cell-Specific Chemotaxis Regulator (ECSCR)
Source: Int J Mol Sci. 2012 Apr 19;13(4):4920–36. doi: 10.3390/ijms13044920 (PMC3344256; doi:10.3390/ijms13044920)
Supplement: Supplementary file 1 [file ijms-13-04920-s001.pdf]

# Supplementary Materials

**Figure S1.** Nucleotide and amino acid sequences of mouse ECSCR isoform-1 precursor. The start codon ATG and the stop codon TAA are underlined.

```
atgcttcgagacatttctctggaagcccacgggctggggtcaacactgacaccccttctt
M  L  R  D  I  S  L  E  A  H  G  L  G  S  T  L  T  P  L  L

gcccatcaactcccacaggggagagtcagaggctacagctctcaacctacgacaactcag
A  H  Q  L  P  Q  G  R  V  R  G  Y  S  S  Q  P  T  T  T  Q

acctcgcaggaaattctacagaagtcttctcaggtctccttggtatccaatcagcctgtg
T  S  Q  E  I  L  Q  K  S  S  Q  V  S  L  V  S  N  Q  P  V

acaccaaggtcaagcaccatggataaacagtccctttccttgcctgacttgatgtccttc
T  P  R  S  S  T  M  D  K  Q  S  L  S  L  P  D  L  M  S  F

cagccacagaagcacacactgggacctggcacaggaaccccagaaaggagcagcagcagc
Q  P  Q  K  H  T  L  G  P  G  T  G  T  P  E  R  S  S  S  S

agcagcagcagcagcagcaggagaggagaagcatctctggatgctactcccagtccagaa
S  S  S  S  S  S  R  R  G  E  A  S  L  D  A  T  P  S  P  E

accaccagccttcagacaaaaaagatgaccatcctgctgaccatcctgcctacccccaca
T  T  S  L  Q  T  K  K  M  T  I  L  L  T  I  L  P  T  P  T

tcagagtcagtgctaactgtggctgcctttggtgtcatcagcttcattgtcatcctggtg
S  E  S  V  L  T  V  A  A  F  G  V  I  S  F  I  V  I  L  V

gttgtagtgatcatcctggtcagtgtggtcagtctaagatttaagtgtcggaagaacaag
V  V  V  I  I  L  V  S  V  V  S  L  R  F  K  C  R  K  N  K

gagtctgaagatccacagaaaccagggagttcagggctgtctgaaagctgctccacagcc
E  S  E  D  P  Q  K  P  G  S  S  G  L  S  E  S  C  S  T  A

aatggagagaaagacagcatcacactcatctccatgaggaacatcaacgtgaacaacagc
N  G  E  K  D  S  I  T  L  I  S  M  R  N  I  N  V  N  N  S

aaaggcagcatgtcagcagagaagattctttaa
K  G  S  M  S  A  E  K  I  L  -
```

**Figure S2.** Nucleotide and amino acid sequences of mouse ECSCR isoform-2 precursor. The start codon ATG and the stop codon TAA are underlined.

```
atggacagagaatacactgaggagcctgctacacacccagctgatctggggaccagcgga
 M  D  R  E  Y  T  E  E  P  A  T  H  P  A  D  L  G  T  S  G

gccatgaggctgggttcagcaattctcggtttactcctgctccaaggctacagctctcaa
 A  M  R  L  G  S  A  I  L  G  L  L  L  L  Q  G  Y  S  S  Q

cctacgacaactcagacctcgcaggaaattctacagaagtcttctcaggtctccttggta
 P  T  T  T  Q  T  S  Q  E  I  L  Q  K  S  S  Q  V  S  L  V

tccaatcagcctgtgacaccaaggtcaagcaccatggataaacagtccctttccttgcct
 S  N  Q  P  V  T  P  R  S  S  T  M  D  K  Q  S  L  S  L  P

gacttgatgtccttccagccacagaagcacacactgggacctggcacaggaaccccagaa
 D  L  M  S  F  Q  P  Q  K  H  T  L  G  P  G  T  G  T  P  E

aggagcagcagcagcagcagcagcagcagcagcaggagaggagaagcatctctggatgct
 R  S  S  S  S  S  S  S  S  S  S  R  R  G  E  A  S  L  D  A

actcccagtccagaaaccaccagccttcagacaaaaaagatgaccatcctgctgaccatc
 T  P  S  P  E  T  T  S  L  Q  T  K  K  M  T  I  L  L  T  I

ctgcctacccccacatcagagtcagtgctaactgtggctgcctttggtgtcatcagcttc
 L  P  T  P  T  S  E  S  V  L  T  V  A  A  F  G  V  I  S  F

attgtcatcctggtggttgtagtgatcatcctggtcagtgtggtcagtctaagatttaag
 I  V  I  L  V  V  V  V  I  I  L  V  S  V  V  S  L  R  F  K

tgtcggaagaacaaggagtctgaagatccacagaaaccagggagttcagggctgtctgaa
 C  R  K  N  K  E  S  E  D  P  Q  K  P  G  S  S  G  L  S  E

agctgctccacagccaatggagagaaagacagcatcacactcatctccatgaggaacatc
 S  C  S  T  A  N  G  E  K  D  S  I  T  L  I  S  M  R  N  I

aacgtgaacaacagcaaaggcagcatgtcagcagagaagattctttaa
 N  V  N  N  S  K  G  S  M  S  A  E  K  I  L  -
```

**Figure S3.** Comparison of nucleotide sequences of the two mouse ECSCR isoform precursors. Identical nucleotides are indicated by asterisks. The start codon ATG and the stop codon TAA are underlined.

```
Isoform-1   ATGCTTCGAGA---CATT------TCT-CTGGAAGCCCA--CGGGCTGGGG-TCAACA-- 45
Isoform-2   ATGGACAGAGAATACACTGAGGAGCCTGCTACACACCCAGCTGATCTGGGGACCAGCGGA 60
            ***    ****   ** *       ** **  *  ****   *  ******  ** *

Isoform-1   ---------CTGA---CACCCCTTCTTGCCCATCAACTCCCACAGGGGAGAGTCAGAGGC 93
Isoform-2   GCCATGAGGCTGGGTTCAGCAATTCTCG---GTTTACTCCTGC---------TCCAAGGC 108
                     ***    ** *  **** *    *  *****  *         **  ****

Isoform-1   TACAGCTCTCAACCTACGACAACTCAGACCTCGCAGGAAATTCTACAGAAGTCTTCTCAG 153
Isoform-2   TACAGCTCTCAACCTACGACAACTCAGACCTCGCAGGAAATTCTACAGAAGTCTTCTCAG 168
            ************************************************************

Isoform-1   GTCTCCTTGGTATCCAATCAGCCTGTGACACCAAGGTCAAGCACCATGGATAAACAGTCC 213
Isoform-2   GTCTCCTTGGTATCCAATCAGCCTGTGACACCAAGGTCAAGCACCATGGATAAACAGTCC 228
            ************************************************************

Isoform-1   CTTTCCTTGCCTGACTTGATGTCCTTCCAGCCACAGAAGCACACACTGGGACCTGGCACA 273
Isoform-2   CTTTCCTTGCCTGACTTGATGTCCTTCCAGCCACAGAAGCACACACTGGGACCTGGCACA 288
            ************************************************************

Isoform-1   GGAACCCCAGAAAGGAGCAGCAGCAGCAGCAGCAGCAGCAGCAGCAGGAGAGGAGAAGCA 333
Isoform-2   GGAACCCCAGAAAGGAGCAGCAGCAGCAGCAGCAGCAGCAGCAGCAGGAGAGGAGAAGCA 348
            ************************************************************

Isoform-1   TCTCTGGATGCTACTCCCAGTCCAGAAACCACCAGCCTTCAGACAAAAAAGATGACCATC 393
Isoform-2   TCTCTGGATGCTACTCCCAGTCCAGAAACCACCAGCCTTCAGACAAAAAAGATGACCATC 408
            ************************************************************

Isoform-1   CTGCTGACCATCCTGCCTACCCCCACATCAGAGTCAGTGCTAACTGTGGCTGCCTTTGGT 453
Isoform-2   CTGCTGACCATCCTGCCTACCCCCACATCAGAGTCAGTGCTAACTGTGGCTGCCTTTGGT 468
            ************************************************************

Isoform-1   GTCATCAGCTTCATTGTCATCCTGGTGGTTGTAGTGATCATCCTGGTCAGTGTGGTCAGT 513
Isoform-2   GTCATCAGCTTCATTGTCATCCTGGTGGTTGTAGTGATCATCCTGGTCAGTGTGGTCAGT 528
            ************************************************************

Isoform-1   CTAAGATTTAAGTGTCGGAAGAACAAGGAGTCTGAAGATCCACAGAAACCAGGGAGTTCA 573
Isoform-2   CTAAGATTTAAGTGTCGGAAGAACAAGGAGTCTGAAGATCCACAGAAACCAGGGAGTTCA 588
            ************************************************************

Isoform-1   GGGCTGTCTGAAAGCTGCTCCACAGCCAATGGAGAGAAAGACAGCATCACACTCATCTCC 633
Isoform-2   GGGCTGTCTGAAAGCTGCTCCACAGCCAATGGAGAGAAAGACAGCATCACACTCATCTCC 648
            ************************************************************

Isoform-1   ATGAGGAACATCAACGTGAACAACAGCAAAGGCAGCATGTCAGCAGAGAAGATTCTTTAA 693
Isoform-2   ATGAGGAACATCAACGTGAACAACAGCAAAGGCAGCATGTCAGCAGAGAAGATTCTTTAA 708
            ************************************************************
```

**Figure S4.** Updated sequences of mouse ECSCR gene. The 9257 bp genomic sequences are based on *Mus musculus* 181000113601716 genomic scaffold, whole genome shotgun sequence (GenBank accession number CH466557.1) (available via www.ncbi.nlm.nih.gov/nuccore/70979735?from=19601084&to=19610340&report=gbwithparts) incorporated with our new data presented here. The mRNA of ECSCR is complement of the sequences by joining 1..346 (Exon 10), 553..649 (Exon 9), 2091..2127 (Exon 8), 2348..2446 (Exon 7), 2878..3000 (Exon 6), 3678..3737 (Exon 5), 4078..4215 (Exon 4), 5273..5311 (Exon 3), 8223..8393 (Exon 2), 9146..9257 (Exon 1). All ten exons are highlighted. The start codon ATG (complement of CAT) in Exon 1 (for splicing variant 2), the start codon ATG (complement of CAT) in Exon 2 (for splicing variant 1), and the stop codon TAA (complement of TTA) in Exon 10 (for splicing variants 1 and 2) are underlined and shown in bold.

1 caggccagaa aataatttta attctagtag aatggacaat ctacctcatt atttccttaa
61 gtcttgctct attgactcct cgttcctgag tttctgagca gcaggaggag ggggagcagg
121 ccaagtgtct agcatggctt ccaagtggga aggagtctct tgtgtggtag gttcttctgg
181 atctagagga atctcatgtc ctttagaact gggagcaggc atgacatcaa ataatttatg
241 tgtggtccgc tgcctccccc aatctcgtct cctctccttc ctcatggcca ggggacagcc
301 gcatcacacg tggacccatg gcgactccag gtcactc**TTA** aagaatctga aatgcatatg
361 tacaatgagg aagggagaac ctgggagggg ccaggtaaga gagcagggga ggaagagcct
421 gggtctgcag ccgtgacctt agcctttctg gctaaggaac cagagcctga aaataggact
481 gaggggtggg ggtgggggt gaggtgagga gtggagtggg tgactgggga gggaagaaca
541 tgtgagatgc accttctctg ctgacatgct gcctttgctg ttgttcacgt tgatgttcct
601 catggagatg agtgtgatgc tgtctttctc tccattggct gtggagcagc tgggatggga
661 aggagccagg gttagagaga agaagggatg gaggaccagg gagtaaaggg gccacggagc
721 actgggcctt cagtcctcat gccagaaaac tctactttgg gatggataat aagaaaatta
781 gaatgtgggc tggtgagatg gctcagcggt taagagcact gactgttctt ccaaaggtcc
841 tgagttcaaa tcccagcaac cacatggtgg ctcacaacta tcggtaatga aatataagga
901 cctcttctgg agtgtctgaa gacagctaca gtatacttac atataacaat aaataaatct
961 ttgggccaga gggagcagag gtactgggtt caattcccag caacctcatg atggcttaca
1021 accatctgta cagctacagt gtacttatat acataaaaga taaataaatc ttaaaaaaaa
1081 aaagaaagaa agaaaatgag aatgtggtct gaaacccacg agacttgtgt ttaaacctca
1141 tttatttctg ttctttttca agctaggttg gtgctttaca accataagcc cagccaggct
1201 gctggtgtac acgtgtaagc ctagcacttg gtaggtaaga ggtaggaaga taggggattc
1261 agggtcatcc tcagctatat aatgagttca aagccagccc aggctctttc tcaacagagc
1321 aaaacaagga ggaagaggag gaggaggagg aggaggaaga ggaggaggag aaacctgttt
1381 ttcctatctt ctagtgtttg atcttgacca agttacaact tgtcctgctc caggttccca
1441 agcatgcttc atgtttcaaa gaagagggaa aaaaatgctg taaagatttt cagtaagaaa
1501 acaaacaaac aaaaaacaaa caaacaaaag attttcagta agatcacaat ttgtatgttg
1561 ttgtgtatgg tatataatag ctttctggtt tggtttttca agacagggtt tctctgtgta
1621 gccctggctg tcctggagct cactctgtag atcagactga ccttgaactc agagatccga
1681 ctgcctctgc ctcccaagtg ctgggattaa aagcatacat caccacaata cctgccttca
1741 gagtaggttt tcaacaagtt tcttcagctt agagaggata tatcttatat cggaatctgt
1801 attttcatga agaggccaca ggagctgagc cctggtgctg aagaccctca cttgtctctg
1861 ctgagcctgg cagcctgctt cagtgacgac tgcagaattg tggggcatat gtcttcaagt
1921 ggtatgaatg ggtatggtat gtggtgggaa gcagaagtgt gtgtgtgtgt gtgtgtgtgt
1981 gtgtgtgtgt gtgtgtgtgt gtgtgtgtac agaaggcaga gagaattgtt ttcctcttgg
2041 ggtaaaggga tttggaatca catttctctc gtgagtttct aaaatcttac ctttcagaca
2101 gccctgaact ccctggtttc tgtggat
[gap 61 bp] Expand Ns
2189 tg tccagcttct cctcagagcc cacccaagct
2221 cacctcccca actcatcaaa tgctgcttct gtgtcattcc ctccctcggc ccagttgcca
2281 tcagcctgag cccccgctca ggctcctgaa gcacaagggt caaagcagcc caggtcaaga

**Figure 4.** *Cont.*

```
2341 caattacctt cagactcctt gttcttccga cacttaaatc ttagactgac cacactgacc
2401 aggatgatca ctacaaccac caggatgaca atgaagctga tgacacctga gcagggaaga
2461 gatgggaaca ggaggaagag gtggaacaat aaggatgagt ctggagttcc ctggcacagg
2521 taagtgcctg tgtgtgtgcg catgcatgtg tgcacgtgtg tatgtgtgtg tgtgtttgtg
2581 tgtgttcgtg tgcacactat atatttgctc tgagagtgaa gtagattctg cttctcttgg
2641 aaacatgagt aatctattag ctggttccct taactaatgt ggaccactta gcccagctac
2701 atcatccata gcaacatgga gcccaagaaa gccaaaaccc ttgcattatg gccttgctta
2761 tcagcaagtc catcccagaa tgaatgggtg agatttgaac ttgcatctta accagagtct
2821 aagtcaatcc tgaggtggag tcccctctca tgccccatcc ccactgccat ctctcaccaa
2881 aggcagccac agttagcact gactctgatg tggggtaggg caggatggtc agcaggatgg
2941 tcatcttttt tgtctgaagg ctggtggttt ctggactggg agtagcatcc agagatgctt
3001 ctgaaataga gtgggtgttg gtgcatcagt gaagggcact gctgagacct ggcaaagaga
3061 atcagcttct ttcctgatgc tctgaggtga gagtttgcac agagctaaca gctcacagaa
3121 aaagactcac tctggacaca accaaccccc tgaagaccga ggatgcagat ctcacatctc
3181 tacatgagta actcggccag ttgctcacat tactgttttc agagaactct agaaaggtta
3241 aaatgcttcc catccccacg tggtacccag aagaaaggat ttatgctgca aaaacaggga
3301 aaccatactg ggcaaagcag agccatctgt gctatgccaa cagttgaaga tgggaggaaa
3361 gaagacattg gcctaaaggg ccagggtgcc aggcaaggga cataggtttc tggccctctt
3421 aaaatgatgt tgggctagct aaatggctca acaggtaaag gtgcttgcaa ccaaagctgg
3481 tagcctaagt tcaatcccta tgacctacat ggttgaagga gaggaccaat gtcacatttt
3541 tcttcccttg cacaagtcaa gtaccctccg gcctattttc taccctattt ccttatttgt
3601 atgagtgtac aatgtccaag aaccaaaaga ggatttaaaa gaaagggtta tagactgaga
3661 acacacactg aacatacctc ctctcctgct gctgctgctg ctgctgctgc tgctgctcct
3721 ttctggggtt cctgtgcctg caccagaaac aaatggccaa gatggaggat cccatctctc
3781 ccaacatact ctgagaaggg tctagccctt tctcaaccct tcccatttcc ctcctccatc
3841 tgttcttttc tcattcccca tcaggagtgt ttaaccttta ctgtgaccag atccaactct
3901 gtttctactt cttcccagtg ctttcttgta atttcccaaa ccagggtacc actgatgtag
3961 tttctagact taagagcctc cactcccaca ctgagggcac cccactaaag gaagtaaaca
4021 cctatccagg catatctagg gcccatcacc tcctgcccac ctgccctgga tactcaccag
4081 gtcccagtgt gtgcttctgt ggctggaagg acatcaagtc aggcaaggaa agggactgtt
4141 tatccatggt gcttgacctt ggtgtcacag gctgattgga taccaaggag acctgagaag
4201 acttctgtag aatttctgtg gataggcaag tacatgtgac cagctcagag tgtcagctat
4261 aatatgcaca gagagtccaa aatacttttt ctttctagtc cttttcttgt gtgtggtgct
4321 agtgattaaa cgcaggatgt ctttcatact gggcaagcac tgtgccccag agctacagcc
4381 acagccctcc tttgtcaacg ccttcaattc ctggaggacc tgttctctgg ctcgcccctg
4441 agattgggaa ggtctttccc tccctccacc ccacagtctc cccttgttca tctcactcct
4501 ttgctggctc gggtctctgg gctgttctca gttcccatag gaagggcccc agagatgacg
4561 tctgaagagg ctgacatcca gatgggccca gtgacacttg ttcctgctcc cttgctctgg
4621 aatcaccagt gtgaggccct gtttgcctgt cccttctgcc acaggctctg catcctcctg
4681 cagagcccaa actgcgtctt gctcctccgg ggcctttgca ggccactatc agcagtctca
4741 gcagctgagt acctagggta acagtgctga gccaaagccc cgggccgcca ggctctgagg
4801 aagatctgtc ttctgcaagg acagagcaga aggggaaagc aaatggggac cccaggatca
4861 agtaggtctg aatggctggg ggtgagcatg ggagggtaga ggtgaccttc tctgcagccg
4921 cacccattca ctaagctgag agaaactagc tggggggctca ggctcctgaa aagccctaaa
4981 acacatcact tgaggggtct gctactctac cctccagccg ttctgtgccc ttctctttgt
5041 ctctgaaggt agatttgttg tttgtttgtt tgttttcctc ccctcctcta cctctgacta
5101 gttatctctt ccctcttcca ggcatgttag gtgaggccag ttgagtgagc ctcagagact
5161 tggcccaagc tcctctcacc tgctttcagt gaaaaagaga gacccgagcc ccctcctcca
5221 ataccttcct aaactcttag gcacagatgc tggagtaggc cccaaatctt accctgcgag
5281 gtctgagttg tcgtaggttg agagctgtag cctgtgagag aaaacaaaag tacacagact
5341 atgaaggggc atggtggcac acatctttaa tcccagaact caggaggcag aagtaggcag
5401 atctctttga attcaaggcc atcctggtct atagagtgag ttccaggaca gccaggacta
5461 catagagaga ccctgtctca aaacaaaaca aaacaagaca aaacaaaaca aaacaaaaca
5521 aaacaaaaca aaacaaaaca aaacaaagac agattgtttt ggtaactgct gagtagagcc
5581 catgagagaa actaggtttt tttggctgtc taaactgtcc atactagaag gcagacacac
```

**Figure 4.** *Cont.*

5641 ttggcaggga attacaggat aagggcttgt caccatcacc tctcagttca gagtcctaga
5701 gtcttctact gtgtcttcag tccttaatag ttatcttagc accctgacac aagtctaggg
5761 gacagttatt caaactagtg acacatctga ccaaacacac agaactctgg gtctctgggt
5821 ctatgcacta ctggtccact tgtttcctcc tgtgaaaact gtcattttgg ctgcaatcta
5881 aaggaagggg agaggaaggt aaatgaaatt gggtagagtt tggggctcgc attgctttgt
5941 gcctgctttc acattctctc tccctctccc tctccctctc cctctccctc ttcctcctc
[gap 459 bp] Expand Ns
6459 cc ttccccactc ccctccctaa
6481 ccagaggtaa tgccctgcct tggaaacaag ttctgaggta tgtccaacag gagaggaagt
6541 gctgaaagga gtgacaggtt taagttaaaa agtggtgttc tagtctaacc ccagggagag
6601 gaggtcactg gcagccactt ttggagctct tgttagctat tctgtttgtt cacatgtgtg
6661 tttgtagcct tggcatagat atgcaatgct tttatgtgtt gggggggggg ggggaggctg
6721 gaaggtcagg ttatgtttgc tcactttcca caatgaaatc attcttgaaa cctatgaaca
6781 atgtgaaagg aaaacaggga aacggaaccg cccatctgga gctgctccct ccacacaggc
6841 tggaactgag gtggccgctc ttttcttcat tagtgcagca tcagacccag gcccctgaag
6901 gactgaggca gctgagttcc aggggcccaa cccaagccgt ggcaacctgc tggtctgttt
6961 gttttcctta aggccttact gttcctccaa ggagtgatcc acactatcca cgtgaaggga
7021 tccacacttg gatactgtgg aattgtttgc aagccaaagc ttacccagcc ctgagatgtg
7081 tttttccaag tcaagatctc tcacattcct gctcctttgt gggtagaggt tccacaaaga
7141 ggtaaatatt acaggaaccc caggctagga aaaacccttt ctagcatggg atgagcctgg
7201 gctaaccctt tgcatccaaa acctctgaag acagttgctt cctgttaaag caccacatac
7261 attccatagt agttctggga ggacttgaag tctgagtgca cctctctaaa tgtatctaga
7321 agatgatctg taagtctttg aggagttccc agggcacaag catcaagctg ggaaggcctg
7381 agctctctgc ctcttatctg actcctccca acttgccatc tctaaaaatt ctttgtaagc
7441 agaggggga cttgaggaat atgttcttct gtaggctggt gcctctgacc caggggacat
7501 acagacaccc agaaagctgt ggccacaaga actcctaact ttgctgacct ctgcttccta
7561 atccctacct gcttcacttc tttccagtct aaccacgccc acgcccctgt tttcctcagc
7621 agcatagttt agggttgaaa agcagcattg gggatgtagc tcagtggtgg agcacttact
7681 tagcatgtgt gagcttgggt gaggccctag gaccaatccc cagcacaaga gaaaaaataa
7741 ataaataaat aaaaagtggg gaatttgagt tctggcttca ctcacagcct gagctggacc
7801 tggcctctct ggaatgtgag actgaggtat aattctgcat aagtcccgtg ctggattctc
7861 agcatcacac cactggggtg gggtgggggga gagcctccga ggttgttgag gggaaaagaa
7921 aatacataca aagcccacag cccctgacac ttgctcaatc ggtgctagat gttgacattt
7981 ttgccccatt ctcttgattt ctccaaatct aaagactggc tgactctcca cagccagaac
8041 ccctgtgttg gcgtgaatgt gactgtctct ctagacagtt cctcagccgc cacaactgct
8101 gacaactgct gtgaatgccc agctgaagaa gttgggcaag ctagggtgcc agtgggttag
8161 acaaagtcta ggctggcatt aggaaggcct gcggaatccc tcataaatcc cccacactaa
8221 cctctgactc tcccctgtgg gagttgatgg gcaagaaggg gtgtcagtgt tgaccccagc
8281 ccgtgggctt ccagagaaat gtctcgaag**C AT**tttctgga acacaaaccc tttctcctaa
8341 gcactctggc ccatcttccc aatacctggt atgcagcctc agatgcagtc cacaaaggac
8401 caggggtggg ggcaggggcg gggccggttg acaaagggaa gttgctgccc tgtgaatcag
8461 agtgtcctgg acttctttct catggttccc agcaatggct ccctaattag aggatcctgc
8521 ctcaagaccc cactcacgac tgccagcgac aatctgagag gtcgaattcc agtcatttca
8581 accagcccct gtgctgtgta tatatcctcc ttggtaaagt gttctttgtg tctaaccaga
8641 aaccctcttg atgtgatttg agattaaagt cagcatttga aattcaaagg ttgtaattct
8701 tcacaggtaa tggcagaaga gaaggtgagc cagaaagccc gaggtgcaga ccattttcgg
8761 ctagaacttt ccatagagtg accatagtgc ctgtatcagt agagtattga cagggcagcc
8821 acagatttct cctgctccta tactccttag aaagaacttt cagggtctgt cattcatcaa
8881 gccaaccact ccccaccttt ctaggggcca gactctatgg gggagctgta gaaaatatcc
8941 taacagcttt acagtctgtt taaccagggt ggtaattccc gtcctccagg ccagcccatc
9001 cctttcatgg aaaggttctg gaatttccct tcttcgggca gagcgttgga tgggaacgtg
9061 ttgggggggg ggggagggca ggagggggaa ggagtgggga ggaggagggg aggcttgctg
9121 gagaaaaccg gcacgtgcat cttaccttgg agcaggagta aaccgagaat tgctgaaccc

**Figure 4.** *Cont.*

```
9181 agcctcatgg ctccgctggt ccccagatca gctgggtgtg tagcaggctc ctcagtgtat
9241 tctctgtcCA Tagtaga
```

© 2012 by the authors; licensee MDPI, Basel, Switzerland. This article is an open access article distributed under the terms and conditions of the Creative Commons Attribution license (http://creativecommons.org/licenses/by/3.0/).
